# Combined pantaloon and femoral hernia: a common pathology in medicine occurring simultaneously

**DOI:** 10.1093/jscr/rjac514

**Published:** 2022-11-19

**Authors:** Karnan Rajkumar, Aakash Trivedi, Ida Molavi, Toghrul Talishinskiy

**Affiliations:** Department of General Surgery, St. Joseph’s University Medical Center, Paterson, NJ 07503, USA; Department of General Surgery, St. Joseph’s University Medical Center, Paterson, NJ 07503, USA; Department of General Surgery, St. Joseph’s University Medical Center, Paterson, NJ 07503, USA; Department of General Surgery, St. Joseph’s University Medical Center, Paterson, NJ 07503, USA

**Keywords:** pantaloon hernia, indirect inguinal hernia, direct inguinal hernia, mesh placement, robotic-assisted repair

## Abstract

Inguinal hernias are typically classified based on their location and can be divided into two types. The most commonly seen inguinal hernias are direct and indirect, which can both potentially require surgical intervention. When both types of hernias are seen simultaneously, it is classified as a pantaloon hernia. This case describes an instance of a femoral hernia being found along with a pantaloon hernia. We present a case of what was projected to be a common inguinal hernia repair but progressed to a rare presentation of a femoral hernia superimposed on a pantaloon hernia. Pantaloon hernias plus a femoral hernia is a rare defect that does not present as often as the different types of isolated hernias.

## INTRODUCTION

There are several types of hernias that are most commonly seen among patients. The inguinal hernias consisting of direct and indirect are typically the ones most frequently seen. However, other hernias exist as well, which include femoral, obturator, umbilical, hiatal and incisional hernias. For inguinal hernias, they are classified based on their anatomic location with respect to the inguinal ligament and inferior epigastric vessels. Hernias presenting medially to the inferior epigastric artery are classified as direct, whereas those lateral are indirect. Indirect hernias have protrusion of abdominal/pelvic contents through the deep and superficial inguinal rings, whereas direct hernias have protrusion only through the superficial inguinal ring. They differ in their etiology as direct hernias are because of a weakness in the transversalis fascia, whereas indirect hernias occur because of a patent processus vaginalis [1]. Nonetheless, these two hernias occurring at the same time are classified as a pantaloon hernia.

Femoral hernias typically occur below the inguinal ligament. This is seen in females as it is an acquired defect because of increases in abdominal pressure or weakness in the pelvic floor, which can be caused by obesity or pregnancy. These have herniation through the femoral ring into the femoral canal and usually occur on the medial aspect of the femoral vein and lateral aspect of the pubic tubercle. A pantaloon hernia and femoral hernia occurring simultaneously are already rare, but having a femoral hernia in a male makes it an even more uncommon presentation.

Inguinal hernias are by far the most common type of hernias worldwide. Other common types of hernia are femoral hernia (6–17%), ventral hernia (3–8.5%) and obturator hernia (<1%) [2]. Reported incidence of pantaloon hernia is 1.6% [3]. The usual presentation for a direct hernia is an older male. Indirect hernias most commonly occur in male infants and older men, whereas femoral hernias are seen in elderly women. The incidence of a pantaloon hernia being found simultaneous to a femoral hernia is not well described in literature. Reducible hernias can be managed non-operatively or surgically corrected on an outpatient basis. Hernias that are incarcerated, strangulated or obstructive need surgical intervention to prevent bowel ischemia and peritonitis. Our patient presented to the emergency department with persistent nausea and abdominal pain worrisome for a complicated inguinal hernia [4].

## CASE REPORT

**Figure 1:**
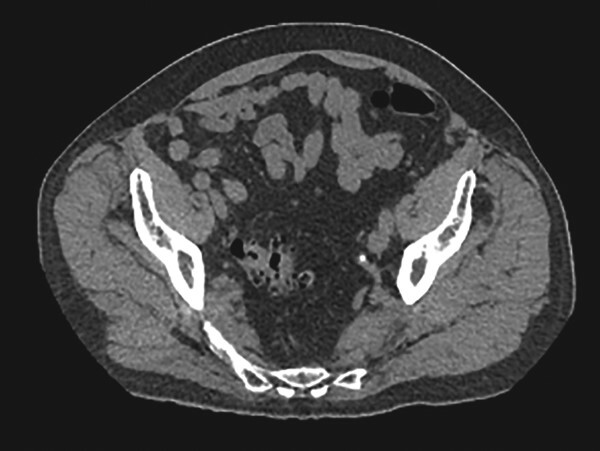
Right inguinal hernia containing bowel.

A 77-year-old male with a past medical history of coronary artery disease presented to the emergency department with right lower quadrant pain associated with constipation and nausea for 2 days. The patient did not have any prior episodes of these symptoms or any previous abdominal surgeries. Physical exam revealed right lower quadrant tenderness. The patient underwent computed tomography of the abdomen and pelvis, which showed diverticulosis and a 5.7 cm AAA. Additionally, it showed a right inguinal hernia containing bowel (Fig. 1). The patient’s symptoms resolved after analgesics and fluid resuscitation. The patient then followed up outpatient for the hernia and was booked for an elective robotic repair.

The patient was taken for a robotic-assisted transabdominal preperitoneal (TAPP) right inguinal hernia repair with mesh placement. Abdominal entry was gained through Veress needle and trocars along with robotic docking was conducted. Attention was then turned to the right side where the peritoneum was incised using electrocautery from the median umbilical ligament laterally. Preperitoneal dissection was taken down to the level of Cooper’s ligament for which there was evidence of direct and femoral defects. The cord structures were identified and then an indirect hernia was also identified. The peritoneum was circumferentially dissected off both the vas deferens and the spermatic cord. The vessels were all protected throughout the entire operation and then an anatomical ProGrip mesh was inserted and placed in an appropriate position. Attention was then turned to closing the peritoneal flap, which was done using a 2–0, 180-day V-Loc. The periumbilical fascia was closed using a 0 Vicryl suture and the skin incisions were closed using 4–0 Monocryl and Dermabond. The patient was discharged to home the same day.

## DISCUSSION

Of the different types of hernias that can occur, pantaloon hernias along with a femoral hernia are very rare occurrences. The patient presented typical abdominal symptoms including nausea, vomiting and constipation. Diagnosis of these concomitant hernias was made at the operation rather than via a clinical diagnosis. Nonetheless, a hernia, especially multiple as in this case, poses a risk of complications such bowel strangulation potentially leading to necrosis and ischemic bowel. Therefore, prompt surgical intervention is warranted when clinical symptoms are indicative.

There are a few approaches to surgically treat a patient presenting with an inguinal hernia, which can be split into an open approach and laparoscopic approach. Open approach includes tissue repairs where the hernia defect is closed with suture. This can be done primarily or with mesh. Laparoscopic approach includes TAPP procedure and total extraperitoneal procedure (TEP), which differ mainly through the approach taken to enter the abdomen. TAPP infiltrates the intra-peritoneum, whereas TEP does not. TAPP can be useful for bilateral hernia repair, large hernia defects and recurrence after open repair. A large mesh can be placed with this approach covering the direct, indirect and femoral spaces [5]. This was the approach used in our case with assistance from the Da Vinci robot. Non-mesh repairs are not recommended due to more chances of recurrence [6]. The optimal approach to fix defects such as this is with mesh especially when there are multiple total defects that need to be corrected. These hernias have the potential to recur and cause similar problems again for the patient. Use of a mesh has been proven to prevent recurrence of these defects. However, the etiology of them needs to be looked into as well. Individuals with inguinal hernias have altered connective tissue compared with controls regarding ratio of collagen fibers, fascia architecture and level of enzymes involved in connective tissue homeostasis [4]. Patient’s with these potential risk factors ought to benefit more from the use of a mesh as their connective tissue dysfunction can lead to recurrence of the herniation. The correct surgical approach is required to prevent all these defects from occurring again.

A femoral hernia is not commonly seen in male patients. A femoral hernia is an uncommon, acquired condition, which has been reported in <5% of all abdominal wall hernias, with a female to male predominance of 1.8:1 [7]. Only a small fraction of hernias that men present with is a femoral hernia. Femoral hernias account for 2% of all hernias in men but they constitute around 24% of all hernias in women [8]. Therefore, it is extremely rare for men to present with femoral hernias. A pantaloon hernia also is not commonly seen and it is an extremely rare presentation when that is coupled with a femoral hernia in a male patient.

## CONCLUSION

Pantaloon hernias alongside a femoral hernia is a unique presentation that does not occur as often as a single hernia would by itself. Also, having a femoral hernia in a male on top of the other two defects is uncommon as well. Therefore, proper surgical intervention is necessary to prevent these defects and potential complications from occurring again.

## CONFLICT OF INTEREST STATEMENT

None declared.

## FUNDING

None.
